# Opioid-induced constipation in patients with cancer pain in Japan (OIC-J study): a post hoc subgroup analysis of patients with lung cancer

**DOI:** 10.1093/jjco/hyaa186

**Published:** 2020-11-07

**Authors:** Hisao Imai, Soichi Fumita, Toshiyuki Harada, Toshio Noriyuki, Makio Gamoh, Masaharu Okamoto, Yusaku Akashi, Yoshiyuki Kizawa, Akihiro Tokoro

**Affiliations:** Division of Respiratory Medicine, Gunma Prefectural Cancer Center, Ota, Japan; Department of Respiratory Medicine, Comprehensive Cancer Center, International Medical Center, Saitama Medical University, Hidaka, Japan; Department of Medical Oncology, Kindai University Nara Hospital, Ikoma, Japan; Center for Respiratory Diseases, JCHO Hokkaido Hospital, Sapporo, Japan; Department of Surgery, Onomichi General Hospital, Onomichi Japan; Department of Medical Oncology, Osaki Citizen Hospital, Osaki, Japan; Medical Affairs, Shionogi & Co., Ltd., Osaka, Japan; Department of Medical Oncology, Kindai University Nara Hospital, Ikoma, Japan; Department of Palliative Medicine, Kobe University Graduate School of Medicine, Kobe, Japan; Department of Psychosomatic Internal Medicine and Supportive and Palliative Care Team, National Hospital Organization Kinki-Chuo Chest Medical Center, Sakai, Japan

**Keywords:** GI-Colorectum-Med, lung-basic, supportive care

## Abstract

**Objective:**

To evaluate the opioid-induced constipation burden in the subgroup of patients with lung cancer who participated in the observational Opioid-Induced Constipation in Patients with Cancer Pain in Japan (OIC-J) study.

**Methods:**

The prospective, observational study, OIC-J, included 212 patients with various tumour types, 33% of whom had lung cancer. The incidence of opioid-induced constipation was evaluated using several diagnostic criteria, as well as the physician’s diagnosis and patient’s subjective assessment. Following initiation of opioids, patients recorded details of bowel movements (i.e. date/time, Bristol Stool Scale form, sensations of incomplete evacuation or anorectal obstruction/blockage and degree of straining) in a diary for 2 weeks. Relationships between patient characteristics and opioid-induced constipation onset and effects of opioid-induced constipation on quality of life were explored.

**Results:**

In total, 69 patients were included in this post hoc analysis. The incidence of opioid-induced constipation varied (39.1–59.1%) depending on which diagnostic criteria was used. Diagnostic criteria that included a quality component or a patient’s feeling of bowel movement as an evaluation item (i.e. Rome IV, physician’s diagnosis, Bowel Function Index, patient’s assessment) showed higher incidences of opioid-induced constipation than recording the number of spontaneous bowel movements alone. Opioid-induced constipation occurred rapidly after initiating opioids and had a significant impact on Patient Assessment of Constipation Symptoms total score (*P* = 0.0031). Patient baseline characteristics did not appear to be predictive of opioid-induced constipation onset.

**Conclusions:**

In patients with lung cancer, opioid-induced constipation can occur quickly after initiating opioids and can negatively impact quality of life. Early management of opioid-induced constipation, with a focus on quality-of-life improvement and patient’s assessments of bowel movements, is important for these patients.

## Teaser

In lung cancer patients, opioid-induced constipation can occur quickly after initiating opioids and can negatively impact quality of life. Treatment with a focus on quality of life and patient’s assessments of bowel movements is important.

## Introduction

Opioid analgesics are the standard of care for moderate-to-severe cancer pain (1–3). Although effective in managing cancer pain, opioid use is often limited by adverse effects, which can lead to their discontinuation due to a significantly negative impact on quality of life (QOL) (4, 5). Opioid-induced constipation (OIC), which is characterized by difficult-to-pass and hard stools, straining at defecation and sensations of incomplete evacuation or anorectal obstruction, is a common side effect of opioid analgesic therapy (6, 7). Criteria for diagnosing OIC have been incorporated into the Rome IV diagnostic criteria for colorectal disorders (8, 9). The Rome IV diagnostic criteria further defines OIC as new or worsening symptoms of constipation when initiating, changing or increasing opioid therapy, and it must include two or more of the following symptoms: straining, lumpy or hard stools, sensation of incomplete evacuation, sensation of anorectal blockage, use of manual manoeuvres to facilitate defecation and fewer than three spontaneous bowel movements (SBMs) per week (8, 9).

The incidence of OIC in patients with cancer widely varies, with reported estimates between 5 and 97%, likely as a result of differing assessment and reporting methods (6, 10), as well as the type of opioid received (11). However, the contribution of other factors, such as cancer type, that may impact the reported incidence of OIC remains unknown. The Opioid-Induced Constipation in Patients with Cancer Pain in Japan (OIC-J) study was a multicenter, prospective, observational study in 212 Japanese patients with cancer pain initiating strong opioids (UMIN000025864) (12). Results of the study demonstrated a rapid onset of OIC (i.e. within 2 weeks of opioid initiation), even with low-dose opioids (mean morphine-equivalent dose, regular use of 19 mg/day), with OIC incidences varying between 45 and 61% depending on the diagnostic criteria used (12). Results from the OIC-J study also demonstrated a strong patient self-awareness of OIC and an effect of OIC on pain management and QOL (13).

The OIC-J study included patients with various tumour types, 33% of whom had lung cancer (12). Every year, there are more than 400 000 deaths in Japan due to cancer, and 20% of those deaths are attributed to lung cancer (14). Patients with lung cancer also frequently experience significant levels of pain, which makes it clinically relevant to better understand pain management strategies in this population (15). Given that patients with lung cancer were well represented in this observational study, this research provided an opportunity to assess whether this particular tumour type had an effect on the incidence of OIC. This post hoc analysis evaluated the incidence of OIC and the relationship between baseline characteristics and OIC onset in a subgroup of patients who had lung cancer and participated in the OIC-J study.

## Methods

### Study design

This post hoc analysis examined data from patients who had lung cancer and were enrolled in the OIC-J study. The OIC-J study (UMIN000025864) was a prospective, observational, cohort study conducted at 28 medical institutions in Japan. The study investigated the incidence of OIC in patients who had cancer pain and were starting strong opioid therapy (12). The study was approved by relevant institutional review boards and conducted in compliance with the Declaration of Helsinki and Ethical Guidelines for Medical and Health Research Involving Human Subjects. All patients provided written informed consent.

### Patients

Inclusion and exclusion criteria of the OIC-J study have been published previously (12). In brief, eligible patients were ≥20 years old, had an Eastern Cooperative Oncology Group performance status (ECOG PS) score ≤2 and had cancer that was expected to be stable for the duration of the study. All patients required the initiation of opioid analgesics and had no constipation (i.e. ≥3 bowel movements during the 7 days prior to enrolment). Patients who had lung cancer and were from the OIC-J study were included in this post hoc analysis. Patients with a history of medical conditions, surgery or radiotherapy that could affect gastrointestinal (GI) structure or function and those who had undergone disimpaction during the 7 days prior to enrolment through to the end of the study period were excluded.

### Endpoints and assessments

The primary endpoint was the incidence of OIC based on Rome IV diagnostic criteria (8). Details of Rome IV diagnostic criteria for a diagnosis of OIC used in this study have been published previously (12). Secondary endpoints included the incidence of OIC based on the attending physician’s diagnosis, occurrence of <3 SBMs (i.e. any bowel movement with the exception of those ≤24 h after rescue laxatives) per week, a Bowel Function Index (BFI) score (16) of ≥28.8 and patient’s daily self-awareness of the presence or absence of OIC symptoms. In addition, the relationship between patient baseline characteristics and OIC onset was explored.

All patients kept a handwritten paper diary for 2 weeks, following initiation of opioids. The date and time of each bowel movement was recorded, as well as the form of stool using the Bristol Stool Scale (17), the presence or absence of the feeling of incomplete evacuation and the degree of straining. In addition, patients rated the sensation of anorectal obstruction/blockage during bowel movements on a scale from 0 (none) to 4 (very severe).

**Table 1:** Patient demographics and baseline clinical characteristics (FAS1 population)

Parameter, n (%)	Patients with lung cancer N = 69
Sex	
Male	55 (80)
Female	14 (20)
Age category, years	
<50	1 (1)
50–64	14 (20)
65–74	26 (38)
≥75	28 (41)
Admission status	
Inpatient	47 (68)
Outpatient	22 (32)
Metastasis	66 (96)
ECOG PS	
0	4 (6)
1	50 (72)
2	15 (22)
Anticancer medications	
Yes	23 (33)
No	46 (67)
Bowel movements in past week	
≥7	19 (28)
7	16 (23)
3–6	34 (49)
<3	0
Rescue laxatives within 24 h before enrolment	3 (4)
Regular laxatives before enrolment	13 (19)
Type of regular-use laxatives	
Magnesium oxide	56 (81)
Naldemedine	7 (10)
Sennosides	3 (4)
Senna	1 (1)
Type of rescue-use laxatives	
Sennosides	16 (23)
Sodium picosulfate	8 (12)
Magnesium oxide	4 (6)
Glycerin	3 (4)
Senna	2 (3)
Lubiprostone	1 (1)
Other	7 (10)
Comorbidities	56 (81)

ECOG PS, Eastern Cooperative Oncology Group performance status; FAS, full analysis set.

**Figure 1.**
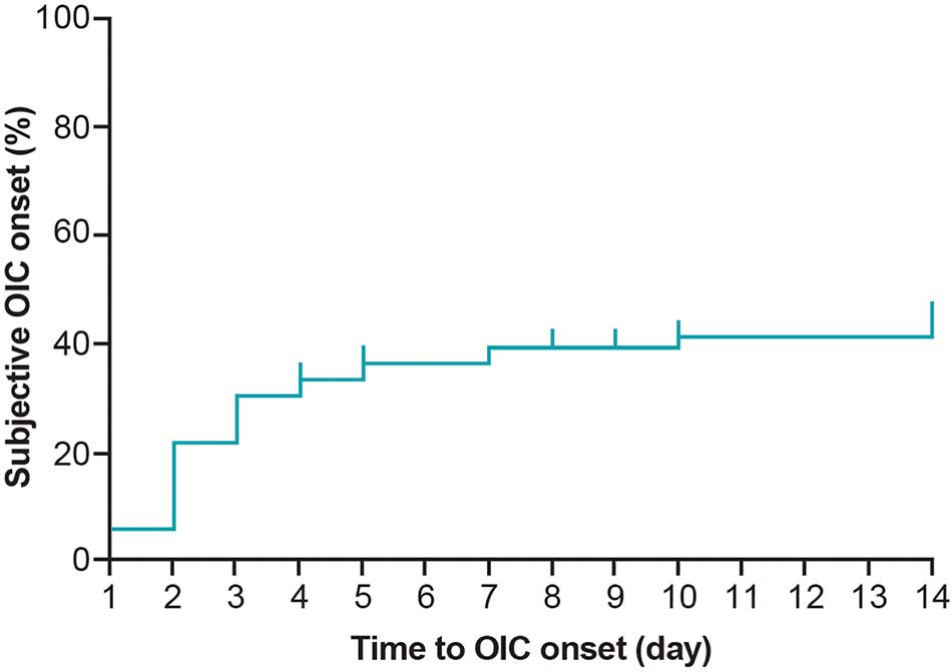
Kaplan–Meier plot of patients’ self-awareness of OIC in patients with lung cancer (FAS1 population). The tick marks in the plot represent censored patients. FAS, full analysis set; OIC, opioid-induced constipation.

Patient self-reported changes in constipation symptoms were assessed using the Patient Assessment of Constipation Symptoms (PAC-SYM) score (18, 19) and QOL was assessed using the Patient Assessment of Constipation Quality-of-Life (PAC-QOL) questionnaire (20). Changes in PAC-SYM and PAC-QOL scores from baseline to 2 weeks after starting opioids were compared between patients with OIC and patients without OIC (as determined by Rome IV diagnostic criteria).

### Statistical analysis

Two populations were defined for the analysis. The full analysis set (FAS) 1 population was defined as all enrolled patients, except those with ethical guideline violations, those with an observation period of <4 days and those who did not take opioids during the observation period. The FAS2 population was defined as all patients in FAS1 with an observation period of ≥7 days.

**Table 2:** Incidence of opioid-induced constipation assessed by various diagnostic criteria (FAS1 population)

Diagnostic criteria	OIC incidence, % (95% CI); N = 69
Rome IV	47.8 (35.6, 60.2)
Physician’s diagnosis	59.1 (46.3, 71.0)
< 3 spontaneous bowel movements per week	39.1 (27.6, 51.6)
Bowel Function Index score ≥ 28.8	53.0 (40.3, 65.4)
Patient assessment	43.5 (31.6, 56.0)

CI, confidence interval; FAS, full analysis set; OIC, opioid-induced constipation.

The incidence of OIC using various diagnostic criteria was calculated as the percentage of patients in the FAS1 population with OIC during the 2 weeks of treatment. The 95% confidence intervals (CIs) were calculated using the Clopper–Pearson method. Associations between patient baseline characteristics and OIC onset were evaluated in the FAS1 population using a contingency table, with *P* values calculated using *χ*^2^ test. Changes from baseline in mean PAC-SYM and PAC-QOL scores were compared between OIC-positive and OIC-negative patients in the FAS2 population using the Welch’s *t*-test. All statistical tests were performed on observed values with a two-sided significance level of 0.05 without multiplicity considerations.

SAS software for Windows, Version 9.4, (SAS Institute Inc., Cary, NC) was used for data analysis.

**Table 3:** OIC incidence according to baseline characteristics (FAS1 population).

	All patients, n	Incidence of OIC, %	95% CI	*P* value^a^
**Primary tumour**				
Lung	69	47.8	35.6, 60.2	–
**Sex**				
Male	55	50.9	37.1, 64.6	0.3096
Female	14	35.7	12.8, 64.9	
**Age, years**				
20–<40	0	–	–	0.2302
40–<50	1	100.0	2.5, 100.0	
50–<65	14	64.3	35.1, 87.2	
65–<75	26	50.0	29.9, 70.1	
≥ 75	28	35.7	18.6, 55.9	
**Admission status**				
Inpatient	47	48.9	34.1, 63.9	0.7873
Outpatient	22	45.5	24.4, 67.8	
**Metastasis**				
No	3	66.7	9.4, 99.2	0.5042
Yes	66	47.0	34.6, 59.7	
**ECOG PS**				
0	4	50.0	6.8, 93.2	0.8795
1	50	46.0	31.8, 60.7	
2	15	53.3	26.6, 78.7	
**Anticancer medications**				
No	46	41.3	27.0, 56.8	0.1251
Yes	23	60.9	38.5, 80.3	
**Bowel movements in past week**				
>7	19	31.6	12.6, 56.6	0.1523
7	16	43.8	19.8, 70.1	
3–6	34	58.8	40.7, 75.4	
<3	0	–	–	
**Rescue laxative within 24 h before study enrolment**				
No	66	45.5	33.1, 58.2	0.0644
Yes	3	100.0	29.2, 100.0	
**Regular-use laxative before study enrolment**				
No	56	50.0	36.3, 63.7	0.4531
Yes	13	38.5	13.9, 68.4	
**Comorbidities**				
No	13	61.5	31.6, 86.1	0.2719
Yes	56	44.6	31.3, 58.5	

^a^ *χ* ^2^ test.

CI, confidence interval; ECOG PS, Eastern Cooperative Oncology Group performance status; FAS, full analysis set; OIC, opioid-induced constipation.

## Results

### Patients

A total of 69 patients with lung cancer, of the 212 patients who had cancer and were enrolled in the OIC-J study, were included in this post hoc analysis and comprised the FAS1 population. Among these patients, 67 patients had follow-up of ≥7 days and were included in the FAS2 population. The majority of patients with lung cancer were elderly (78% were ≥65 years old), were not receiving anticancer medications (67%), had metastatic disease (96%) and had comorbidities (81%) at baseline (Table 1). A total of 4 and 19% of patients who had lung cancer received rescue laxatives and regular-use laxatives, respectively, 24 h prior to enrolment.

Oxycodone was the most common opioid used (n = 61), followed by morphine (n = 8), fentanyl (n = 3) and hydromorphone (n = 3). The mean (SD) morphine equivalent daily dose of opioid analgesic taken by patients with OIC or without OIC was 22.3 (14.7) mg/day and 20.0 (8.9) mg/day, respectively.

### Incidence of OIC

The incidence of OIC varied depending on which diagnostic criteria was used (Table 2). The incidence of OIC was higher (43.5–59.1%) when using assessments that included a quality component or patient’s feeling of bowel movement (i.e. Rome IV diagnostic criteria, physician’s diagnosis, BFI or patient’s subjective assessments) compared with assessment of SBM, which resulted in an OIC incidence of 39.1% (95% CI 27.6, 51.6). The patient-assessed incidence of OIC was 43.5% (95% CI 31.6, 56.0; Online Resource 1) 2 weeks after starting opioids (40.3% after the first week). The onset of OIC after initiation of opioids, according to patient’s subjective assessments, was rapid (Fig. 1). No significant associations were observed between patient baseline characteristics and OIC onset (Table 3).

### Effect of OIC on QOL

**Figure 2.**
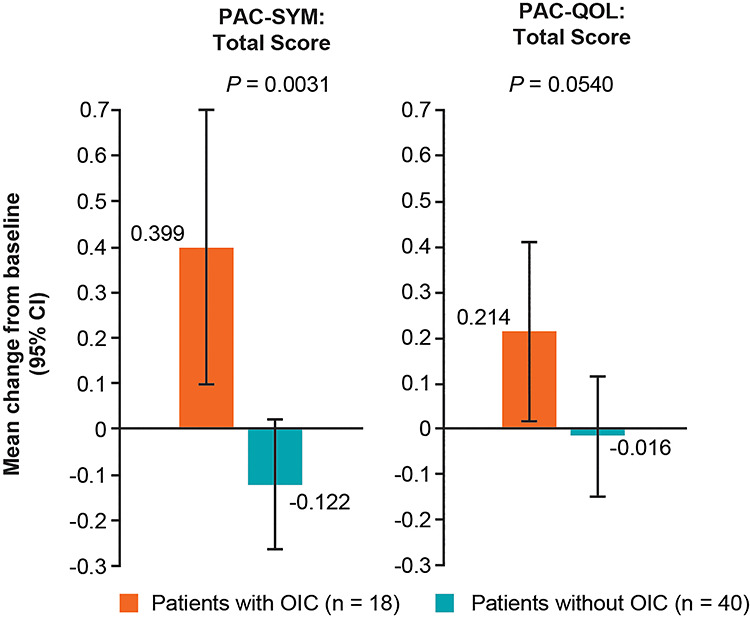
Mean changes from baseline in PAC-SYM and PAC-QOL total scores in patients with lung cancer and OIC versus patients with lung cancer and no OIC (FAS2 population); *P* values were calculated using the Welch’s *t*-test. CI, confidence interval; FAS, full analysis set; OIC, opioid-induced constipation; PAC-QOL, Patient Assessment of Constipation Quality of Life; PAC-SYM, Patient Assessment of Constipation Symptoms.

Among 67 patients in the FAS2 population, 58 patients completed the PAC-SYM and PAC-QOL questionnaires. Of these patients, 18 were given an OIC diagnosis and 40 were not, based on Rome IV diagnostic criteria. A comparison of patients with or without OIC demonstrated a significant difference in the mean change from baseline in PAC-SYM scores (+0.399 [95% CI 0.097, 0.701] vs −0.122 [95% CI −0.264, 0.020], respectively; *P* = 0.0031) 2 weeks after initiating opioid therapy (Fig. 2). The mean change from baseline for the PAC-SYM subscores of abdominal symptoms (AS) and stool symptoms (SS) were also significantly different between patients with or without OIC (AS, +0.343 [95% CI 0.004, 0.681] vs −0.063 [95% CI −0.235, 0.110], respectively; *P* = 0.0343; SS, +0.467 [95% CI 0.034, 0.900] vs −0.241 [95% CI −0.471, −0.110], respectively; *P* = 0.0054) **(**Online Resource 2**)**.

The mean change from baseline in the PAC-QOL scores for patients with and without OIC (+0.214 [95% CI 0.015–0.413] vs −0.016 [95% CI −0.150–0.118]; *P* = 0.0540) was numerically higher 2 weeks after initiating opioid therapy **(**Fig. 2**)**. There was a significant difference in the mean change from baseline for the subscore of psychosocial discomfort between patients with or without OIC (+0.208 [95% CI −0.002, 0.418] vs −0.038 [95% CI −0.141, 0.066]; *P* = 0.0371) (Online Resource 2).

## Discussion

Results of this post hoc analysis of patients with lung cancer showed that the incidence of OIC varied (39.1–59.1%) depending on which diagnostic criteria was used. Use of Rome IV diagnostic criteria, physician’s diagnosis, BFI, or patient’s subjective assessment, which all include a quality component or a patient’s feeling of a bowel movement as an evaluation item, showed higher incidences of OIC than recording the number of SBMs alone. The results of this analysis demonstrated that OIC occurs rapidly after initiating opioids in patients with lung cancer, consistent with findings from the primary analysis of the OIC-J study. A significant difference in total scores between patients with OIC compared with patients without OIC was observed for PAC-SYM, but not for PAC-QOL. However, the mean change from baseline in PAC-QOL total scores was numerically higher in patients with OIC compared with patients without OIC. Changes in PAC-QOL total score have been positively correlated with changes in PAC-SYM total score (21), and constipation symptoms are known to affect QOL (22). Taken together, OIC in patients in this population can affect QOL based on PAC-SYM and PAC-QOL total scores.

Patient baseline characteristics did not appear to be associated with the onset of OIC. The incidence of OIC in patients who received anticancer medication was numerically higher than patients who did not receive anticancer medication (60.9% vs 41.3%, respectively). GI symptoms, including constipation, are a burden for a large proportion of patients receiving palliative treatment for cancer. These types of symptoms may arise either from the disease itself or from side effects of treatment, particularly cancer chemotherapy (23). Therefore, it is possible that some patients in the anticancer medication group may have experienced chemotherapy-induced constipation, contributing to the observed difference. Chemotherapy data were not collected as a part of this study, precluding additional analysis for patients who received prior anticancer medication.

The incidences of OIC in patients with lung cancer were similar to those reported in the overall population of patients in the OIC-J study, which varied from 45% (SBM) to 61% (physician’s diagnosis) (12). In contrast, an independent post hoc analysis of patients from the OIC-J study demonstrated higher incidences of OIC in a subgroup of patients with GI cancers (24). This result may be due, in part, to physicians and patients having an increased focus on GI symptoms.

These results support the findings of the primary OIC-J study, underscoring the importance of early OIC management focusing on QOL and patients’ feelings of bowel movement in patients with lung cancer. European Society of Medical Oncology guidelines for the assessment and management of constipation in patients with cancer acknowledge that some diagnostic criteria do not adequately consider the patient’s subjective experience of constipation, which can lead to under-recognition and under-treatment (11). These guidelines suggest that measurable objective symptoms (e.g. stool characteristics, defecation frequency), patient perception, level of discomfort and ease of defecation should be taken into consideration when diagnosing constipation (11). Constipation and diarrhoea have been reported as common side effects of many of the chemotherapy agents used to treat lung cancer (23), and one-third of the patients in the current analysis were also receiving anticancer medications.

This appears to be the first study to publish data regarding the incidence of OIC, specifically in patients with lung cancer. Additional observational studies specifically evaluating the incidence of OIC in patients with lung cancer are lacking. While valuable, the analysis is limited by the relatively small number of patients and the post hoc design.

## Conclusions

In patients with lung cancer, OIC can occur quickly after the initiation of opioid therapy and can negatively impact QOL. Early management of OIC, with a focus on QOL improvement and the patient’s subjective assessment of bowel movements, is important for these patients.

## Funding

This work was supported by Shionogi & Co., Ltd., Osaka, Japan. Medical writing and editorial assistance were funded by Shionogi & Co., Ltd.

## Conflicts of Interest

**Hisao Imai** has received travel reimbursement from Shionogi. **Soichi Fumita** has received honoraria from Merck Serono, Ono Pharmaceutical Co., Bristol Myers Squibb, Takeda and Bayer Yakuhin, and travel reimbursement from BeiGene. **Toshiyuki Harada** has received honoraria from Taiho Pharmaceutical, AstraZeneca K.K., Boehringer Ingelheim and Hisamitsu Pharmaceutical Co., Inc. **Toshio Noriyuki** has received personal fees from Shionogi, Taiho Pharmaceutical and Chugai Pharmaceutical, and non-financial support from Shionogi. **Makio Gamoh** had received honoraria from Taiho Pharmaceutical, Chugai Pharmaceutical, Yakult Honsha, Ono Pharmaceutical, Takeda, Daiichi-Sankyo, Nippon Kayaku and Eli Lilly Japan. **Masaharu Okamoto** was an employee of Shionogi at the time that this work was conducted. **Yusaku Akashi** has received honoraria from AstraZeneca K.K., Chugai Pharmaceutical Co., Ltd., MDS K.K., Bristol Myers Squibb K.K., Nippon Boehringer Ingelheim Co., Ltd, Pfizer Seiyaku K.K., Taiho Pharmaceutical Co., Ltd. and Hisamitsu Pharmaceutical Co., Inc. **Yoshiyuki Kizawa** has received grants from Shionogi, Kyowa-Kirin and Terumo, and personal fees from Shionogi, Kyowa Kirin Terumo, Johnson & Johnson, Daiichi-Sankyo, Pfizer, Mundipharma and Chugai Pharmaceutical. **Akihiro Tokoro** has received grants from Shionogi and Daiichi-Sankyo, and personal fees from Daiichi-Sankyo, Mundipharma, Hisamitsu, Terumo, Sumitomo Dainippon and Kracie.

## Author contributions

Hisao Imai, Soichi Fumita, Toshiyuki Harada, Toshio Noriyuki, Makio Gamoh, Masaharu Okamoto, Yusaku Akashi, Yoshiyuki Kizawa and Akihiro Tokoro contributed to the study conception and design of the analysis. Data collection, analysis and interpretation were performed by Hisao Imai, Soichi Fumita, Toshiyuki Harada, Toshio Noriyuki, Makio Gamoh, Masaharu Okamoto, Yusaku Akashi, Yoshiyuki Kizawa and Akihiro Tokoro. All authors critically reviewed all drafts of the manuscript and approved the final manuscript for submission.

## Additional information

Co-author Makio Gamoh is deceased.

## Supplementary Material

Supplemental_Figure_1_hyaa186

Online_Resource_1_hyaa186
