# Supplementary figures and images for: Opioid-induced constipation in patients with cancer pain in Japan (OIC-J study): a post hoc subgroup analysis of patients with lung cancer
Source: Jpn J Clin Oncol. 2020 Nov 7;51(3):444–50. doi: 10.1093/jjco/hyaa186 (PMC7937418; doi:10.1093/jjco/hyaa186)

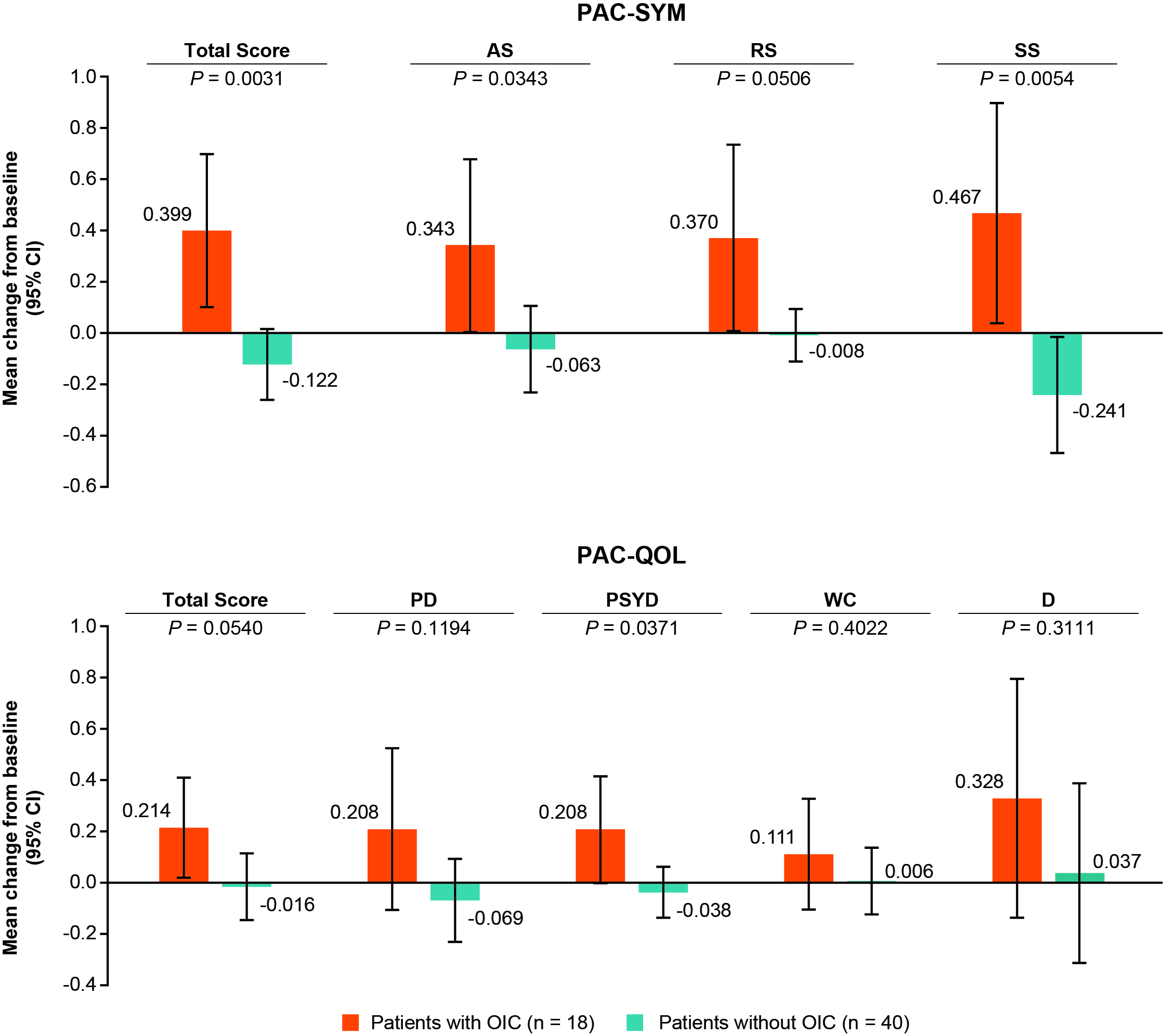

Supplement: Supplemental_Figure_1_hyaa186 [file supplemental_figure_1_hyaa186.jpeg]
