# Supplementary material for: Opioid-induced constipation in patients with cancer pain in Japan (OIC-J study): a post hoc subgroup analysis of patients with lung cancer
Source: Jpn J Clin Oncol. 2020 Nov 7;51(3):444–50. doi: 10.1093/jjco/hyaa186 (PMC7937418; doi:10.1093/jjco/hyaa186)
Supplement: Online_Resource_1_hyaa186 [file online_resource_1_hyaa186.docx]

**Online Resource 1.** Incidence of OIC awareness (FAS1 population)

| **N** | **OIC Awareness, n** | **Incidence of OIC Awareness (%)** | **95% CI (Clopper-Pearson)** |
| --- | --- | --- | --- |
| 69 | 30 | 43.5 | 31.6–56.0 |

CI, confidence interval; FAS, full analysis set; OIC, opioid-induced constipation.
